# Caveolin-1 modulates intraocular pressure: implications for caveolae mechanoprotection in glaucoma

**DOI:** 10.1038/srep37127

**Published:** 2016-11-14

**Authors:** Michael H. Elliott, Nicole E. Ashpole, Xiaowu Gu, Leonie Herrnberger, Mark E. McClellan, Gina L. Griffith, Alaina M. Reagan, Timothy M. Boyce, Masaki Tanito, Ernst R. Tamm, W. Daniel Stamer

**Affiliations:** 1 Department of Ophthalmology/Dean McGee Eye Institute, University of Oklahoma Health Sciences Center, Oklahoma City, 73104, OK, USA; 2 Department of Ophthalmology/Duke Eye Center, Duke University, Durham, 27710, NC, USA; 3 Institute of Human Anatomy and Embryology, Universität Regensburg, 93053 Regensburg, Germany; 4 Division of Ophthalmology, Matsue Red Cross Hospital, Matsue, Shimane 690-8506 Japan

## Abstract

Polymorphisms in the *CAV1/2* genes that encode signature proteins of caveolae are associated with glaucoma, the second leading cause of blindness worldwide, and with its major risk factor, intraocular pressure (IOP). We hypothesized that caveolin-1 (Cav-1) participates in IOP maintenance via modulation of aqueous humor drainage from the eye. We localize caveolae proteins to human and murine conventional drainage tissues and show that caveolae respond to mechanical stimulation. We show that Cav-1-deficient (Cav-1^−/−^) mice display ocular hypertension explained by reduced pressure-dependent drainage of aqueous humor. Cav-1 deficiency results in loss of caveolae in the Schlemm’s canal (SC) and trabecular meshwork. However, their absence did not appear to impact development nor adult form of the conventional outflow tissues according to rigorous quantitative ultrastructural analyses, but did affect cell and tissue behavior. Thus, when IOP is experimentally elevated, cells of the Cav-1^−/−^ outflow tissues are more susceptible to plasma membrane rupture indicating that caveolae play a role in mechanoprotection. Additionally, aqueous drainage from Cav-1^−/−^ eyes was more sensitive to nitric oxide (NO) synthase inhibition than controls, suggesting that excess NO partially compensates for outflow pathway dysfunction. These results provide a functional link between a glaucoma risk gene and glaucoma-relevant pathophysiology.

Glaucoma is a heterogeneous group of diseases that collectively are the second leading cause of blindness worldwide, with primary open angle glaucoma (POAG) being the most prevalent form^1^. Intraocular pressure (IOP) is the principal risk factor for POAG. The conventional outflow pathway, consisting of the trabecular meshwork (TM) and a specialized, drainage vessel called Schlemm’s canal (SC)^2^, controls IOP by varying resistance to aqueous humor outflow in response to IOP fluctuations. This pathway is the pathological location of ocular hypertension in POAG^3^. Despite the fact that IOP is generated by conventional outflow resistance, IOP-lowering drugs that target this pathway are only beginning to be realized^3^. Furthermore, the molecular mechanisms that control pressure-dependent outflow are not well understood, but mechanotransduction in TM and SC cells appears critical^4^,^5^,^6^,^7^.

Caveolae, specialized membrane domains, are abundant in the SC and TM of the conventional outflow pathway^8^,^9^. Gene association studies have reproducibly associated polymorphisms at *CAV1/2* gene loci with both POAG and IOP^10^,^11^,^12^,^13^,^14^,^15^,^16^,^17^,^18^. However, functional role(s) that caveolins/caveolae play in IOP maintenance are incompletely understood. We have previously found retinal functional deficits and vascular pathologies in mice deficient in Cav-1^19^,^20^ but the associations of these with glaucomatous injury are unknown. Caveolae are implicated in mechanoprotection^21^,^22^,^23^,^24^,^25^ and mechanotransduction^26^,^27^ and transduce flow-mediated vasodilation by endothelial nitric oxide synthase (eNOS)^28^, an important mediator of IOP^5^,^29^. As the conventional outflow pathway tissues that maintain IOP homeostasis are subject to large mechanical loads, we hypothesized that caveolae serve a critical function as membrane mechanosensors/protectors in the pressure-dependent aqueous humor drainage.

In the present study, we examined whether Cav-1 is an important endogenous modulator of IOP and aqueous humor outflow. We provide evidence that caveolae components are abundant in conventional outflow tissues and that caveolae respond to mechanical stimulation. Caveolae deficiency results in IOP elevation, reduced aqueous humor drainage, and increased sensitivity of Cav-1-deficient outflow pathway cells to rupture from acute IOP elevation. Morphological and quantitative ultrastructural analyses reveal that Cav-1 deficiency results in loss of caveolae from the conventional outflow tract without significant structural defects; suggesting that the functional deficits in IOP maintenance and outflow facility do not result from developmental abnormalities in the outflow pathway. The results presented demonstrate important roles for caveolae in outflow pathway function and imply that caveolae participate in mechanoprotection of outflow pathway cells, *in vivo*.

## Results

### Localization of caveolae proteins to human and murine conventional outflow tissues and *in vitro* evidence of mechanically-induced caveolae disassembly in human outflow pathway cells

**Figure 1:**
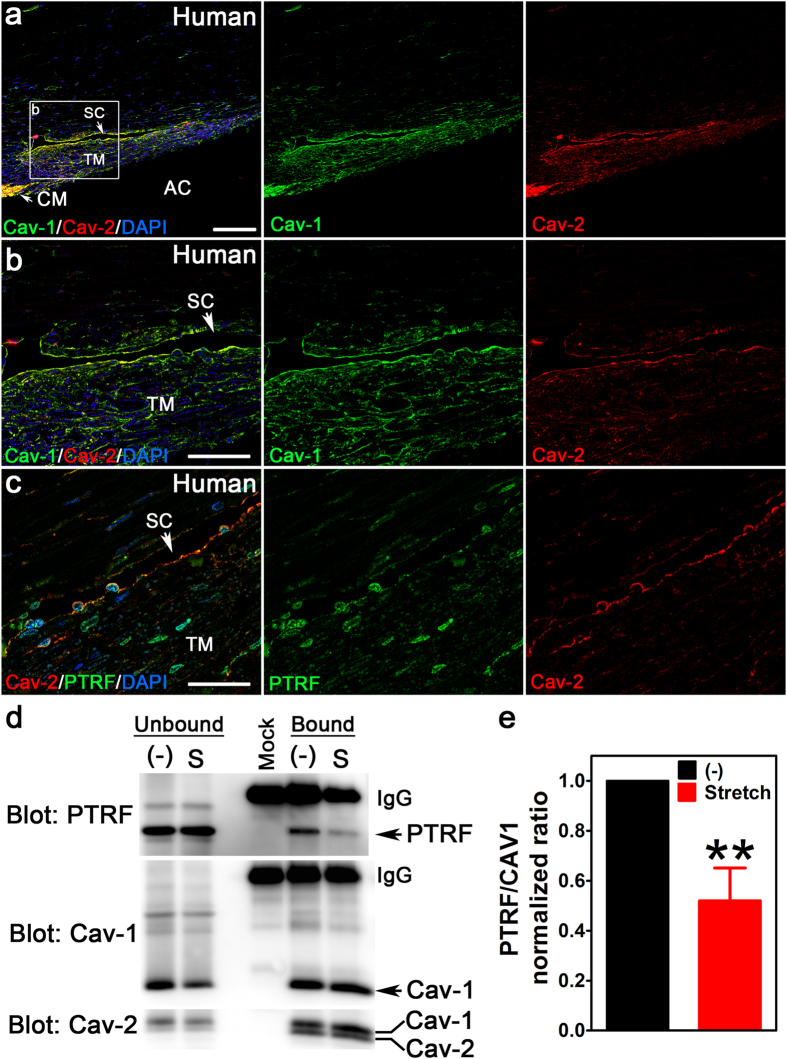
Caveolae proteins localize to human outflow tissue and respond to mechanical stress in human TM cells. (**a,b**) Cav-1 (green) and Cav-2 (red) colocalize in the TM and SC of the human conventional outflow pathway. The white box in (**a**) delineates the region shown at higher magnification in (**b**). *Abbreviations:* TM, trabecular meshwork; SC, Schlemm’s canal; CM, ciliary muscle; AC, anterior chamber. (**c**) PTRF/cavin-1 (green), a protein essential for caveolae formation, partially localizes with Cav-2 (red) but is also present in nuclei of the TM and SC. Scale bars = 100 μm in (**a**), 50 μm in (**b**), and 30 μm in (**c**). (**d,e**) Stretch-induced dissociation of Cav-1 and PTRF/cavin-1 was assessed by coimmunoprecipitation using Cav-1 antibody for immunoprecipitation. Cyclic stretch (15%, 1 Hz, for 24 h) resulted in reduced recovery of PTRF/cavin-1 with Cav-1 (upper panel) with no change in the association of Cav-2 (lower panel). Lane labeled “Mock” represents the caveolin-1 antibody incubated without cell lysate to indicate position of IgG and any bands resulting from the antibody alone. (**e**) Densitometric analysis of the stretch-induced dissociation of PTRF/cavin-1 and Cav-1 (**p ≤ 0.01, one sample t-test compared to theoretical mean of 1 (normalized control), n = 4 independent cell strains). *Abbreviations:* “(−)”, no stretch; “S”, cyclic stretch.

The conventional outflow pathway controls pressure-dependent aqueous humor drainage from the eye and is the primary location of glaucoma pathology^3^. The two outflow cell types, the TM and SC endothelium, contain morphologically-identifiable caveolae^8^,^9^. We first identified whether molecular components of caveolae, Cav-1, Cav-2, and “polymerase I and transcript release factor” (PTRF/cavin-1), are localized in human conventional outflow tissues. We labeled iridocorneal angle sections from human eyes with antibodies against caveolae proteins and found that Cav-1 and Cav-2 colocalized in the SC, TM, and ciliary muscle (CM) (Fig. 1a,b). PTRF/cavin-1 partially colocalized with Cav-2 in SC and TM but also localized to DAPI-positive nuclei in SC and TM cells (Fig. 1c).

As conventional outflow pathway cells are normally subjected and responsive to fluctuating mechanical loads^3^, we determined whether caveolae, putative mechanosensors/protectors^21^,^22^,^23^,^24^,^25^,^26^,^27^,^28^,^30^, respond to mechanical stimulation in human TM cells subjected to a model of cyclic mechanical stretch that mimics ocular pulse fluctuation. Association of PTRF/cavin-1, which is essential for caveolae formation and dissociates from Cav-1 during caveolae disassembly^21^,^30^, was assessed by co-immunoprecipitation. As shown in Fig. 1d,e, cyclic stretch resulted in significantly reduced interaction of PTRF/cavin-1 with Cav-1 but the complex between Cav-1 and Cav-2 was not affected by mechanical perturbation. These results demonstrate that caveolae respond to mechanical stimulation in outflow pathway cells, *in vitro*, and imply that caveolae sense mechanical perturbations due to fluctuating IOP. We thus set out to determine the consequences of Cav-1 ablation on outflow pathway function, *in vivo*.

**Figure 2:**
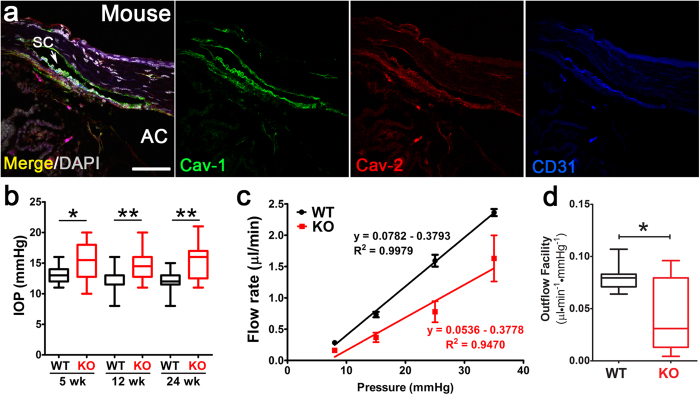
Loss of Cav-1 results in increased IOP and reduced aqueous humor drainage. (**a**) Localization of Cav-1 (green) and Cav-2 (red) in murine outflow pathway is similar to that observed in human tissue. CD31 (blue) labels the SC endothelium. (**b**) Intraocular pressure was significantly increased in *Cav1*^−/−^ mice compared to controls as measured by rebound tonometry at indicated ages (*p ≤ 0.05, ** p ≤ 0.01, one-way ANOVA and Newman-Keuls *post hoc* analysis, n = 13–22 mice for each group). (**c**) Conventional, pressure-dependent outflow is significantly reduced in *Cav1*^−/−^ eyes. Outflow facility was measured in perfused, enucleated eyes subjected to sequential pressure steps. Pressure-flow relationships (*left panel*) for *Cav1*^−/−^ and control eyes. The slope of the regression line (conventional outflow facility) is fit through the mean ± SEM values for n = 8 control and n = 9 *Cav1*^−/−^ eyes (from n = 5 control and n = 6 *Cav1*^−/−^ mice. (**d**) Comparison of conventional outflow facility between *Cav1*^−/−^ and control eyes (*p ≤ 0.05, unpaired two-tailed t-test).

As the murine conventional outflow pathway is structurally and functionally analogous to its human counterpart^31^,^32^, we compared the localization of caveolae proteins in control and *Cav1*^−/−^ mouse outflow pathways. As observed in human tissue, we detected Cav-1 and Cav-2 immunoreactivity in murine SC and TM (Fig. 2a). Cav-2 immunoreactivity was reduced in Cav-1-deficient tissue (Supplementary Fig. 1) confirming that Cav-1 ablation downregulates Cav-2 as observed in non-ocular tissues^33^. These results establish *Cav1*^−/−^ mice as a model to investigate the roles of Cav-1 in outflow physiology and IOP maintenance.

### Caveolin-1 deficiency results in increased intraocular pressure, reduced pressure-dependent outflow, without significant structural changes in the murine conventional outflow pathway

Given the localization of caveolae proteins to the conventional outflow pathway, the association of the *CAV1/CAV2* gene locus with POAG^10^,^11^, and evidence that caveolae in TM cells respond to mechanical stimulation, we reasoned that loss of Cav-1 would impact IOP, the primary risk factor in POAG. We compared IOP in *Cav1*^−/−^ mice to controls using non-invasive, rebound tonometry. Since Cav-1 is a negative regulator of eNOS^28^ and eNOS overexpression reduces IOP^5^, we hypothesized that Cav-1 loss reduces IOP via secondary eNOS hyperactivity. Interestingly, we observed the opposite; significant ocular hypertension in Cav-1-deficient mice that was sustained at several ages (Fig. 2b). The difference in IOP between *Cav1*^−/−^ and control mice was 2–3 mmHg at all ages examined and did not significantly increase with age.

To determine whether ocular hypertension in Cav-1^−/−^ mice resulted from increased resistance (decreased facility) to aqueous humor drainage, we measured flow while holding at sequential pressures in perfused Cav-1^−/−^ and control eyes as previously described^5^,^32^. The pressure-flow curves shown in Fig. 2c indicate that Cav-1 loss resulted in a significant 42% reduction of pressure-dependent outflow (the slope of the linear pressure-flow curve; Fig. 2d). In Cav-1^−/−^ mice, it is important to note that the flow rate at high pressure deviated from the linear pressure-flow relationship such that the conventional outflow facility was further reduced when the highest pressure was removed (0.036 ± 0.009, mean ± SEM) without affecting control facility (0.078 ± 0.005, mean ± SEM). These results indicate that loss of Cav-1 dramatically impairs pressure-dependent aqueous humor drainage and that this reduction in outflow facility explains the 2–3 mmHg IOP differential between *Cav1*^−/−^ and control mice.

**Figure 3:**
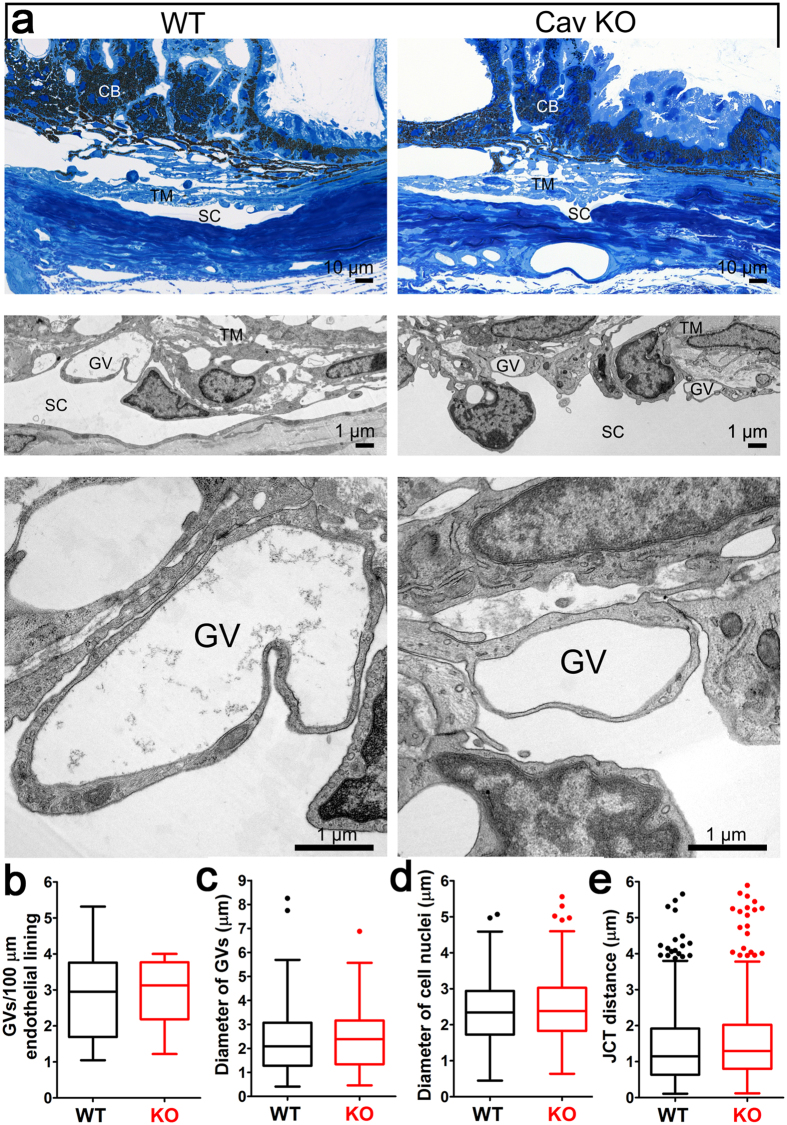
Morphological and ultrastructural analyses of Cav-1^−/−^ conventional outflow pathway. (**a**) Semithin sections (Richardson’s stain) through the iridocorneal angle of representative control (*upper left panel*) and Cav-1^−/−^ (*upper right panel*) eyes. *Middle and lower panels* in (**a**) show higher magnifications by transmission electron microscopy (TEM). The chamber angle is open in control and Cav-1^−/−^ eyes while obvious abnormalities of ciliary body (CB), trabecular meshwork (TM), and Schlemm’s canal (SC) are absent. At higher magnification (*middle* and *lower panels*), giant vacuoles (GV) are detectable in both genotypes. (**b–e**) Quantitative ultrastructural analyses of control and Cav-1^−/−^ eyes. No significant differences in the number of GVs (**b**) or their sizes (**c**) were observed. (**d**) Endothelial nuclei diameter, a measurement of endothelial thickness, was not significantly different between genotypes. (**e**) The JCT depth was also not significantly different between Cav-1^−/−^ and control eyes. Eyes from 4 Cav-1^−/−^ mice and 7 littermate controls were used for these quantitative analyses. Numbers of GVs were quantified in 86 non-overlapping images from Cav-1^−/−^ eyes and 151 images from controls. Giant vacuole diameter measurements were made on 74 Cav-1^−/−^ and 107 control GVs. Endothelial cell nuclei diameters were measured from 118 Cav-1^−/−^ and 155 control nuclei. The depth of the JCT was measured at seven locations in each image for a total of 490 and 735 measurements in Cav-1^−/−^ and control eyes, respectively. For a schematic on how these measurements were made, see Supplementary Fig. 2.

Given that Cav-1 ablation resulted in significant reductions in pressure-dependent outflow, we examined outflow pathway morphology in Cav-1^−/−^ and control eyes. By light microscopy, we observed no gross abnormalities in the iridocorneal angle, ciliary body, iris or cornea (Fig. 3a). As ultrastructural abnormalities might provide an explanation for the functional deficits in the *Cav1*^−/−^ outflow pathway, we proceeded with a rigorous quantitative analysis of outflow pathway ultrastructure (Fig. 3b–e). We examined four quadrants/eye at superior, inferior, nasal, and temporal locations from eyes from n = 7 wildtype and n = 4 *Cav1*^−/−^ mice as described in the methods (see also Supplemental Fig. 2 for a schematic of how measurements were performed). We hypothesized that caveolae are necessary for the formation of giant vacuoles (GVs), which are large endothelial outpouchings into the SC lumen that are a hallmark feature of the normal SC inner wall (see Fig. 3) and form as a result of the pressure gradient in the basal to apical direction across the endothelium^9^,^34^. As expected, a consistent ultrastructural finding was the complete absence of morphologically-identifiable caveolae. However, outflow pathway ultrastructure showed a high degree of regional, within-eye variability. Thus, we could not identify significant differences in GV numbers or diameter between wildtype and *Cav1*^−/−^ eyes (Fig. 3b,c). We also examined endothelial nuclei diameter (Fig. 3d) and juxtacanalicular connective tissue (JCT) depth (Fig. 3e) to determine whether outflow tissues were altered in *Cav1*^−/−^ eyes. Neither parameter was found to be significantly different between genotypes. These results indicate that caveolae deficiency does not result in significant alterations in outflow pathway morphology or ultrastructure. Thus, the functional deficits observed in *Cav1*^−/−^ mice cannot be explained by gross changes in outflow pathway morphology and likely do not result from developmental abnormalities in the SC or TM. These results also highlight the high degree of location-dependent variability in outflow pathway morphology and the need for rigorous quantitative analysis of outflow pathway ultrastructure.

### Caveolae protect the outflow pathway from acute intraocular pressure elevation, *in vivo*

**Figure 4:**
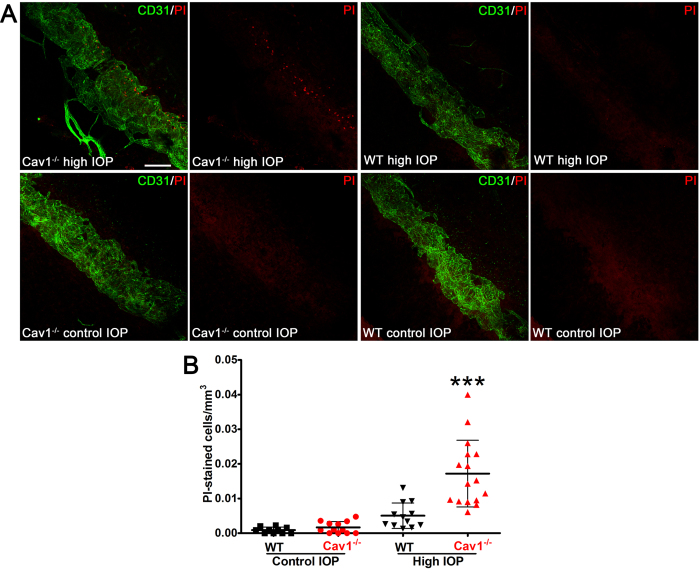
Caveolae protect SC endothelial membranes from IOP-induced damage. (**A**) Representative maximum projections of z-stacks from anterior segment wholemounts that encompass the entire SC diameter from Cav-1^−/−^ and littermate controls subjected to IOP elevation (upper panels) or maintained at standing IOP (lower panels). The SC endothelium was co-stained with CD31 (green) and PI-stained nuclei are labeled in red. Scale bar = 100 μm. (**B**) Quantification of PI-stained nuclei in each image stack (from n = 5 mice/genotype). For statistical analysis, multiple images from each eye were averaged to yield a single value of PI cells/mm^3^ per mouse. ***p ≤ 0.001, one-way ANOVA and Newman-Keuls *post hoc* analysis.

The SC endothelium inner wall (facing the anterior chamber) is subjected to transient, fluctuating stretch throughout the day. Under pressure, the formation of GVs routinely increase in the area of the inner wall by 50%^35^ resulting in large-scale perturbations of the endothelial membrane. As caveolae are abundant features of the SC endothelium that participate in mechanosensation and mechanoprotection^21^,^22^,^23^,^24^,^25^,^26^,^27^,^28^, we hypothesized that caveolae donate membrane to buffer SC inner wall stretch during GV formation. Thus, caveolae-deficient outflow cells may have compromised ability to resist pressure-induced fluctuations in mechanical load across the SC inner wall. An intriguing parallel is recent evidence that caveolae-deficient vascular endothelial cells are sensitive to mechanical rupture in response to increased cardiac output^21^. To test this idea, *in vivo*, we modified the labeling strategy of Cheng *et al.*^21^ to assess nuclear incorporation of the membrane-impermeant dye, propidium iodide (PI), in outflow tissue subjected to acute IOP elevation. High IOP resulted in a significant increase in PI-labeled nuclei in Cav-1^−/−^ compared to control SC (Fig. 4A,B) suggesting enhanced sensitivity to acute membrane damage. Most PI-labeled cells were located within the SC (as delineated by CD31 immunostaining) and tended to be closer to the anterior chamber at the location of the SC inner wall. However, some PI-positive cells were just adjacent to the SC suggesting the possibility that caveolae-deficient TM cells may also have ruptured. The absence of PI staining in eyes not subjected to IOP elevation indicates that PI incorporation in Cav-1^−/−^ SC cells resulted from acute membrane rupture and rules out cell death prior to IOP increase.

**Figure 5:**
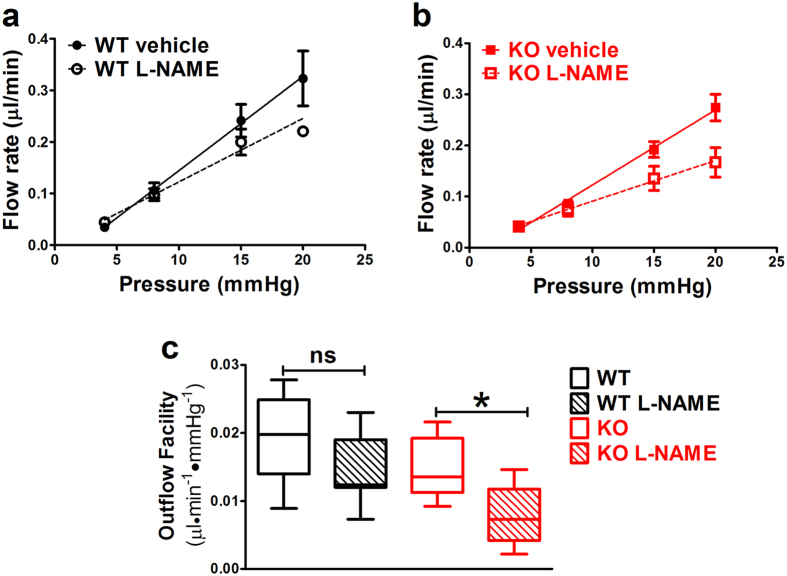
*Cav1*^−/−^ eyes are more sensitive to NOS inhibition than control eyes. Outflow facility was measured in paired eyes perfused with 10 μM L-NAME or PBS vehicle in control (**a**) and *Cav1*^−/−^ (**b**) eyes. (**c**) *Cav1*^−/−^ eyes treated with L-NAME had significant reduction in conventional outflow facility compared to control eyes (p ≤ 0.05, one-way ANOVA with Newman-Keuls *post hoc* test, n = 7–9). Outflow facilities (mean ± SEM) were 0.019 ± 0.003 for control + vehicle, 0.014 ± 0.002 for control + L-NAME, 0.015 ± 0.002 for *Cav1*^−/−^ + vehicle, and 0.008 ± 0.001 for *Cav1*^−/−^ + L-NAME.

Our results suggest that at least one function of Cav-1/caveolae in the outflow pathway is membrane mechanoprotection during IOP transients. However, in addition to membrane donation, caveolae disassembly can also transduce mechanical signals^23^,^27^,^28^. Cav-1 is an established negative regulator of eNOS^28^ and Cav-1^−/−^ mice display increased eNOS activity^36^ and reduced ability to activate eNOS in response to shear stress^28^. As eNOS activity reduces IOP and increases pressure-dependent drainage^5^, it was surprising that Cav-1^−/−^ eyes had increased IOP and outflow resistance. We hypothesized that eNOS hyperactivity resulting from caveolae deficiency partially compensates for Cav-1-dependent SC defects and thus, Cav-1^−/−^ eyes would be more sensitive to eNOS inhibition. We perfused Cav-1^−/−^ and control eyes with the eNOS inhibitor, N-ω-nitro-L-arginine methyl ester (L-NAME) or vehicle while measuring pressure-dependent outflow. As shown in Fig. 5a–c, L-NAME significantly reduced outflow facility by 45% in Cav-1^−/−^ eyes compared to vehicle-perfused eyes similar to recently published results^29^. Importantly, L-NAME reduced outflow facility in wildtype control eyes by only 26%, consistent with our previous report^37^. These results demonstrate enhanced sensitivity of Cav-1^−/−^ eyes to eNOS inhibition and that eNOS hyperactivity secondary to Cav-1 loss may partially rescue outflow pathology.

## Discussion

In the current study, we provide compelling mechanistic evidence that Cav-1 and caveolae are critical modulators of IOP and aqueous humor drainage and that the presence of caveolae protects the SC endothelium from IOP elevation and mediates NO signaling. Several functions attributed to caveolins/caveolae likely impact IOP and aqueous outflow including membrane mechanosensation/mechanoprotection, regulation of eNOS activity, transcellular transport/transendothelial pore formation, regulation of matrix remodeling, and cytoskeletal dynamics^23^,^38^,^39^. Recently, transient knockdown of caveolins in the TM was shown to influence matrix remodeling and to increase outflow facility^40^. In contrast, our findings and those of two independent recent reports^29^,^41^, demonstrated reduced outflow facility in chronically caveolae-deficient mice. Thus, it appears that chronic loss of Cav-1 and caveolae results in functional changes in the conventional outflow pathway that are not induced by short-term transient silencing of Cav-1. Transient partial silencing may induce short-term cellular responses to reductions of Cav-1, such as rapid eNOS hyperactivation which could transiently increase outflow facility^5^. Interestingly, transient silencing of *CAV2* did reduce outflow facility in this previous study^40^. As Cav-2 is not essential for caveolae formation and requires Cav-1 for stabilization of its expression, Cav-1 deficiency leads to concomitant reductions in Cav-2^33^. Our findings (Supplementary Fig. 1) support this observation and indicate that Cav-2 does not compensate for Cav-1 loss in outflow tissue.

Mechanical stimulation of outflow pathway cells results in dissociation of PTRF/cavin-1 from Cav-1 (Fig. 1d,e). This is consistent with prior studies demonstrating that endothelial caveolae disassemble in response to mechanical perturbation and that caveolae act as mechanosensors and membrane reservoirs to buffer biomechanical stresses^21^,^22^,^23^,^24^,^25^,^26^,^27^,^28^,^30^. As caveolae are absent from Cav-1-deficient outflow tissue and pressure-dependent outflow facility is reduced in such tissue, these results collectively demonstrate a critical role for caveolae in the ability of outflow tissue to sense and respond to fluctuating IOP. Although caveolae deficiency does not result in gross alterations in outflow pathway ultrastructure (Fig. 3) at baseline IOP, we provide compelling evidence that caveolae-deficient outflow tissue is more sensitive to cellular rupture in response to experimental IOP elevation (Fig. 4). The acute IOP elevation that resulted in cell rupture in our experimental paradigm is higher than that normally observed *in vivo*. However, brief and transient IOP spikes approaching or exceeding 50 mmHg have been observed by continuous IOP monitoring in human and non-human primates^42^,^43^,^44^. Based on limitations in our experimental strategy, we did not examine whether exposure of short term IOP transients might also result in cellular damage in the caveolae-deficient background. Intraocular pressure elevation results in the formation of GVs via partial detachment of SC endothelium from its underlying extracellular matrix and considerable increases in endothelial surface area^35^. Thus, membrane donation by caveolae disassembly may be an important mechanism by which outflow tissue is protected from membrane damage during GV formation resulting from chronic IOP elevation or acute IOP spikes. We have previously shown that outflow pathway cells are also protected from mechanical strain by the aquaporin-1 water channel^45^ emphasizing the dynamic mechanical environment of the outflow tract. Furthermore, the partial localization of aquaporin-1 to caveolae in a variety of non-ocular cells^46^,^47^,^48^, suggest the possibility that caveolae-dependent mechanoprotection is mediated by aquaporin-1. Moreover, as GVs are frequent sites of transendothelial pores that facilitate aqueous humor flow through the SC inner wall^9^, caveolae deficiency may result in reduced pore density. Reduced pore density is an intrinsic feature of glaucomatous SC both *in situ* and in culture^4^,^49^,^50^,^51^ and likely contributes to increased resistance to aqueous humor outflow. Biomechanical strain induces pore formation in SC cells and caveolae-mediated membrane fusion events may produce “mini-pores” that provide nucleating centers for pore formation^49^. In support of this idea, plasmalemmal vesicle-associated protein (PLVAP), the diaphragm protein for caveolae and fenestrae is also a structural feature of GV minipores^8^,^9^. Given the relatively greater abundance of caveolae versus mini-pores in the SC, it is likely that most of the PLVAP found in the SC is derived from caveolae as previously suggested^8^. Thus, it is intriguing to speculate that caveolae ablation may reduce pore formation which could explain the increased IOP and reduced pressure-dependent outflow in Cav-1-deficient mice. However, given the paucity of minipores identified in ultrathin sections from control SC, we cannot address this question from our quantitative TEM analysis.

Besides the passive role of membrane donation by caveolae to protect the SC from biomechanical stress, caveolae-mediated mechanical activation of eNOS may influence outflow facility. It is well-established that caveolae are critical for shear-mediated eNOS activation in vascular endothelium^28^. As eNOS activation reduces IOP and increases conventional outflow^5^, it is possible that the loss of caveolar mechanotransduction in Cav-1-deficient eyes affects the ability of the outflow pathway to increase outflow via eNOS activation. We were initially surprised that loss of Cav-1, which is an endogenous inhibitor of eNOS^52^, results in elevated IOP because eNOS hyperactivity is an established feature of Cav-1-deficient mice^36^,^53^ and would be predicted to reduce IOP. We thus speculated that eNOS hyperactivity secondary to Cav-1 ablation compensates for some other functional deficit imparted on outflow physiology by caveolae deficiency. Our results using the eNOS inhibitor, L-NAME support this idea as outflow facility was more dramatically affected by eNOS inhibition in Cav-1-deficient eyes than in wildtype controls (Fig. 5). The effect of L-NAME on Cav-1^−/−^ outflow facility was recently reported^29^ but surprisingly no direct comparison was made to wildtype controls in this study. This study also reported that topical application of a NOS inhibitor (L-NAME) increased IOP while application of a NO donor (sodium nitroprusside) decreased IOP. However, the effect on IOP was similar between Cav-1^−/−^ and wildtype mice, suggesting lack of enhanced sensitivity. In contrast, our experiments provide direct evidence of enhanced sensitivity to eNOS inhibition in caveolae-deficient outflow tissue and agree with a previous study on Cav-1-associated pulmonary hypertension where pulmonary arterial resistance was more dramatically increased by eNOS inhibition in Cav-1^−/−^ than in control mice^54^. In their recent report, Lei *et al.*^29^ suggest that hyperactive eNOS in the Cav-1 null background enhances production of NO-derived oxidants that damage the outflow pathway. However, whether nitrative stress is the mechanism by which caveolae deficiency induces ocular hypertension remains to be mechanistically tested.

It is currently unclear whether POAG-associated *CAV1* polymorphisms result in reduced outflow tract caveolae although some polymorphisms are associated with reduced caveolin-1 expression in non-ocular tissues^12^,^18^. If, in fact, caveolae are reduced in the outflow tract in POAG, one would predict that eNOS hyperactivity secondary to caveolae loss would partially compensate for IOP elevation. This begs the question of whether POAG patients with caveolae deficiency would benefit from NO donors currently under development as IOP lowering therapies^55^. Although not addressed directly in our study, the recent results of Lei *et al.*^29^ show that application of a NO donor has similar IOP-lowering effects on Cav1-deficient and Cav1-competent mice and that NO was able to increase outflow facility in the Cav1 null background. As complete Cav1 deletion results in total ablation of caveolae in the outflow tract, it follows that NO donors could provide therapeutic benefit in the context of caveolae deficiency. It remains to be determined empirically if other IOP lowering therapies are effective in the face of caveolae deficiency.

In conclusion, our results provide novel insight into how a glaucoma-associated gene product impacts IOP and pressure-dependent aqueous humor drainage. Our data support the hypothesis that caveolae provide membrane platforms to both buffer mechanical strain resulting from fluctuations in IOP and mechanically transduce signals (e.g., eNOS activation) that alter the resistance properties of the outflow tissues. We predict that caveolae deficiency renders the outflow tissue sensitive to repetitive pressure fluctuations that lead to pathological changes in the outflow pathway that reduce aqueous humor drainage. While the link between *CAV1/2* polymorphisms, IOP, and POAG remain to be determined, our results showing the consequences of caveolae deficiency on IOP and aqueous outflow indicate that caveolae are essential contributors to IOP maintenance. As some glaucoma-associated *CAV1/2* polymorphisms result in reduced expression of caveolin-1 in non-ocular tissues^12^,^18^, our results suggest that reduced caveolae could impact disease pathogenesis. Thus, our studies establish an important relationship between caveolae and cellular processes at the level of the inner wall that appear important in the regulation of outflow resistance and tissue homeostasis. Caveolae appear to provide a “membrane buffer” for the inner wall in response to IOP elevations, which occur in all eyes during normal daily activities^42^. Due to these critical roles in outflow function, caveolae represent a viable target for therapeutic development in POAG.

## Methods

### Animals

Experiments were performed on wildtype and Cav-1^−/−^ mice in the C57BL/6 J background^19^,^20^,^56^. Mice were bred and maintained in the specific pathogen-free rodent barrier facility at the University of Oklahoma Health Sciences Center and were transferred to biocontainment caging in the conventional vivarium at the Dean McGee Eye Institute prior to experimentation. This study has been conducted in compliance with the Animal Welfare Act, the implementing Animal Welfare Regulations, and the principles of the Guide for the Care and Use of Laboratory Animals. All procedures were approved by the Institutional Animal Care and Use Committee of the University of Oklahoma Health Sciences Center and were carried out in accordance with the ARVO Statement for the Use of Animals in Ophthalmic and Vision Research.

### Human Tissues and Primary Human TM Cells

Human eye tissue sections and primary human TM cells used in these studies were prepared from cadaver eyes acquired from the Iowa Lions Eye Bank (Coralville, IA) and Miracles in Sight Eye Bank (Winston-Salem, NC) who obtain informed consent from donor families. Both eye banks are accredited by the Eye Bank Association of America. All eyes are de-identified prior to receipt in the laboratory. Use of tissue and cells from these de-identified cadaver eyes was determined to not constitute human subjects research by the Institutional Review of the University of Oklahoma Health Sciences Center.

### Co-Immunoprecipitation of Caveolae Proteins from Mechanically-Stimulated Human TM Cells

Primary human TM cells were isolated from cadaver eyes as previously described^57^. Cells were seeded on type I collagen-coated Flexcell plates (Flexcell International Corp., Burlington, NC) as previously described^58^. Cell stretching (15% stretch) was performed at frequency of 1 Hz, mimicking the ocular pulse, for 24 h in serum-free DMEM using the FX-3000 Flexercell Strain Unit (Flexcell International Corp., Burlington, NC). Control cells were placed on the Flexercell Strain Unit but were not subjected to cyclic mechanical stress. Control and stretched cells were lysed in Tris-buffered saline buffer containing 60 mM octyl glucoside and protease inhibitors (EMD Millipore, Billerica, MA), precleared by centrifugation and immunoprecipitated with rabbit polyclonal anti-Cav-1 antibody (BD Pharmingen, San Diego, CA) as previously described^19^,^59^. Bound and unbound proteins were resolved by SDS-PAGE on 4–20% polyacrylamide gels and electrotransferred to nitrocellulose membranes for immunoblotting with rabbit polyclonal PTRF/cavin-1 antibody (1:1000, EMD Millipore, Billerica, MA). Blots were reprobed sequentially with rabbit polyclonal anti-Cav-1 and mouse monoclonal anti-Cav-2 (1:500, clone 65, BD Pharmingen, San Diego, CA). Immunoreactivity was detected using horseradish peroxidase-conjugated secondary antibodies and West Dura chemiluminescent substrate (Life Technologies, Grand Island, NY) on a Carestream *In Vivo* F-Pro imaging system (Carestream, Rochester, NY).

For densitometric analysis, immune complexes from four independent cell strains from stretched and unstretched control cells were blotted as described and band intensities for PTRF/cavin-1 and Cav-1 were measured using Carestream *In Vivo* F-Pro software. Band intensities (arbitrary units) for both PTRF/cavin-1 and Cav-1 from stretched samples were normalized to that of the control sample on each independent blot then the normalized ratio of PTRF/cavin-1 to Cav-1 was determined. These normalized ratios were then compared to a theoretical mean of 1 (the normalized control ratio) by one-way t-test using GraphPad Prism software.

### IOP Measurements

We measured IOP in one eye of male and female mice (5 weeks to 6 months of age) immediately after loss of consciousness following intraperitoneal ketamine (100 mg/kg)/xylazine (10 mg/kg) by a commercially available rodent rebound tonometer (TonoLab; ICare, Espoo, Finland) as previously described and per manufacturer’s instructions^5^. Pressures were consistently measured between 9 am and noon, central time (2–5 h after lights-on in the vivarium).

### Outflow Facility Measurements

Mice were euthanized by CO_2_ inhalation followed by cervical dislocation at the end of the day (between 6–7 pm, Central Time) and eyes were immediately placed in DMEM (Life Technologies, Grand Island, NY) containing either high or low glucose. High glucose media was used in early experiments (Fig. 2), and was switched to the more physiological low glucose for later experiments shown in Fig. 5. Eyes were shipped (FedEx, Priority Overnight) from Oklahoma City, OK to Durham, NC for early morning arrival (between 7:30–8:30 am, Eastern Time). Upon arrival, eyes were immediately cannulated with a 33-gauge beveled-tip needle (Nanofil; World Precision Instruments, Sarasota, FL) and perfused as previously described^5^,^32^. The perfusion cannula was connected to a glass syringe (50 μL; Hamilton, Reno, NV) and a pressure transducer (model 142PC01G; Honeywell, Morristown, NJ). A variable flow rate was delivered to the anterior chamber via a computer-controlled syringe pump (model PHD 2000, Harvard Apparatus, Holliston, MA) to maintain a desired IOP as monitored by the computer-controlled pressure transducer (Labview Software; National Instruments, Austin, TX). Depending on experiment, eyes were held sequentially at the pressures of either 8, 15, 25 and 35 mmHg (Fig. 2) or 4, 8, 15 and 20 mmHg (Fig. 5); and the perfusion duration was at least 15 min at each pressure step. Note: Refinements in the perfusion protocol dictated the change in pressure steps for the second set of experiments shown in Fig. 5. In some experiments, one eye was perfused with DBG (PBS (Gibco by Life Technologies, Grand Island, NY) supplemented with 5.5 mM glucose (Sigma-Aldrich, St. Louis, MO)) and the paired contralateral eye, with DBG containing 10 μM L-NAME (Sigma-Aldrich, St. Louis, MO) at the same sequential pressure steps. The order (left versus right and drug versus vehicle) in which eye perfusions were performed was randomized. Total facility (*C*_total_) was calculated as *C*_total_ = total inflow rate (*F*)/intraocular pressure (IOP), where we assumed that at equilibrium, total inflow rate equals the total outflow rate and episcleral pressure is 0 mmHg for enucleated eyes. Conventional, pressure-dependent outflow was estimated using linear regression analysis of the pressure/flow relationship.

### Propidium iodide labeling of acute cell rupture

*In vivo* PI labeling was based on a modification of the protocol recently developed by Cheng *et al.*^21^ to label vascular endothelial cell rupture. Instead of infusing intravenously, we prepared a 20 μg/ml PI solution in sterile saline and filled a fluid reservoir connected to an anterior chamber infusion cannula (30 g needle). Intraocular pressure of Cav-1^−/−^ and control eyes was experimentally increased to 50 mmHg by elevating the PI-filled reservoir for 30 min in anesthetized mice. We chose this perfusion pressure based on a prior study showing that a pressures of 50 mmHg induces the formation of large GVs in the SC^34^. Immediately following acute IOP elevation, mice were euthanized, eyes were dissected, fixed with 4% paraformaldehyde, and anterior segment wholemounts were prepared as described^2^. Wholemounts were labeled with CD31 to identify the SC in confocal slices imaged on an Olympus FV1200 confocal microscope. Propidium-iodide labeled nuclei were quantified using Image J (Rasband, W.S., ImageJ, U. S. National Institutes of Health, Bethesda, Maryland, USA, http://imagej.nih.gov/ij/, 1997–2015) and SC volume from image slices was determined using the VolumEst plug-in (http://lepo.it.da.ut.ee/~markkom/volumest/).

### Immunohistochemistry, Histology, and Ultrastructural Analysis

For immunohistochemistry, eyes enucleated from mice euthanized by CO_2_ inhalation were fixed by immersion in Prefer fixative (Anatech Ltd., Battle Creek, MI) and processed for paraffin sectioning as previously described^56^. Human corneal rim tissue from cadaver eyes were also fixed with Prefer and processed for paraffin sectioning as described. The use of these tissues for this study was determined to be exempt by the Institutional Review Board at OUHSC. Immunohistochemistry was performed as previously described^20^,^56^. Briefly, 5 μm sections were deparaffinized, washed with PBS containing 0.1% Triton X-100 (PBST) and blocked with 10% normal horse serum in PBST. To block endogenous IgG in mouse tissues labeled with mouse monoclonal antibodies, sections were also blocked with 25 μg/ml monovalent anti-mouse Fab (Jackson Immunoresearch Laboratories, Inc., West Grove, PA) in PBS for 1 h at room temperature followed by one wash with PBS prior to incubation with primary antibodies. The following primary antibodies were incubated overnight at 4 °C: rabbit anti-Cav-1 (1:200; BD Biosciences, San Diego, CA); mouse anti-Cav-2 (1:100; BD Biosciences); and rabbit anti-PTRF/Cavin-1 (1:100; EMD Millipore, Billirica, MA). Sections were incubated with appropriate fluorophore-conjugated secondary antibodies (1:500 Life Technologies or Jackson Immunoresearch Laboratories, Inc.) for 1–2 h at room temperature. Nuclei were counterstained with DAPI (Sigma-Aldrich). Imaging was performed using a FV1200 confocal laser scanning microscope (Olympus, Tokyo, Japan) and images were processed with Photoshop CS5 (Adobe Systems, San Jose, CA).

For light microscopy and transmission electron microscopy of mouse eyes^8^, mice were deeply anesthetized with ketamine (100 mg/kg)/xylazine (10 mg/kg) and fixed by intracardiac perfusion of Karnovsky’s fixative [2% glutaraldehyde, 2% paraformaldehyde, 0.02% CaCl_2_ in 0.1 M cacodylate buffer (pH 7.2)]. After fixation, the eyes were sent to Germany in cacodylate buffer. Postfixation was accomplished in a mixture of 1% OsO_4_ and 0.8% potassium ferrocyanide in 0.1 M cacodylate buffer for 2 h at 4 °C. Specimens were then dehydrated in a graded series of ethanol and embedded in Epon (Serva, Heidelberg, Germany). Semithin sections (1 μm) were collected on uncoated glass slides and stained with methylene blue⁄azure II after Richardson *et al.*^60^ for light microscopy. Ultrathin sections were mounted on uncoated copper grids, stained with uranyl acetate and lead citrate and examined on a Zeiss EM 10 A electron microscope (Zeiss, Oberkochen, Germany).

For quantitative transmission electron microscopy (TEM) seven wildtype mice and four Cav^−/−^ littermates were perfused through the heart with 2% paraformaldehyde/2% glutaraldehyde in cacodylate buffer. Both eyes from each animal were enucleated, immersed in the same fixative and sent to Germany for analysis. Prior to enucleation, the nasal quadrant of each eye was marked by cauterization. One eye from one wildtype animal was ruptured during enucleation and excluded from the quantitative analysis. Prior to embedding each eye was dissected into four (superior, inferior, nasal and temporal) quadrants and embedded in Epon as described above. From the anterior eye segment of each quadrant at least one meridional semithin and ultrathin section was cut and examined by light microscopy or TEM, respectively. Ultrathin sections were mounted on slot grids to allow visualization of the total SC length. For each eye, the number of giant vacuoles per 100 μm endothelial length was determined. A first qualitative analysis had indicated the presence of smaller size giant vacuoles and the presence of thick SC endothelial cells protruding into the lumen of SC in mutant mice. Therefore, as additional parameters, the largest diameter of each vacuole counted was measured as was the diameter of SC endothelial nuclei in the SC-JCT direction that were present on the same sections (Supplementary Fig. 2). Finally, the depth of the JCT was analyzed by measuring the distance between outer membrane of SC cell to inner membrane of the next JCT cell in SC-JCT direction and perpendicular to the direction of the SC endothelial lining (Supplementary Fig. 2). Six measurements were taken at a distance of 1 μm from each other and averaged. Statistical analysis was done by a two-tailed Student’s test and *p* values ≤ 0.05 were considered to be statistically significant. Semithin sections were stained with Richardson’s stain and analyzed on an Axiovision microscope (Carl Zeiss). Ultrathin sections were investigated on a Zeiss Libra transmission electronmicroscope. Measurements were done software-assisted (Axiovision software 3.0, Carl Zeiss).

### Statistics

Graphing and statistical analyses were performed using GraphPad Prism v.5 (GraphPad Software, La Jolla, CA). P values ≤ 0.05 were considered significant and data are presented as mean ± SEM.

## Additional Information

**How to cite this article**: Elliott, M. H. *et al.* Caveolin-1 modulates intraocular pressure: implications for caveolae mechanoprotection in glaucoma. *Sci. Rep.* **6**, 37127; doi: 10.1038/srep37127 (2016).

**Publisher’s note**: Springer Nature remains neutral with regard to jurisdictional claims in published maps and institutional affiliations.

## Supplementary Material

Supplementary Information
