# Supplementary material for: Caveolin-1 modulates intraocular pressure: implications for caveolae mechanoprotection in glaucoma
Source: Sci Rep. 2016 Nov 14;6:37127. doi: 10.1038/srep37127 (PMC5107904; doi:10.1038/srep37127)
Supplement: Supplementary Information [file srep37127-s1.pdf]

Supplementary Information

Caveolin-1 modulates intraocular pressure: implications for caveolae mechanoprotection in glaucoma

Michael H. Elliott[1]*, Nicole E. Ashpole[2], Xiaowu Gu[1], Leonie Herrnberger[3], Mark E. McClellan[1], Gina L. Griffith[1], Alaina M. Reagan[1], Timothy M. Boyce[1], Masaki Tanito[4], Ernst R. Tamm[3], and W. Daniel Stamer[2]

[1]Department of Ophthalmology/Dean McGee Eye Institute, University of Oklahoma Health Sciences Center, Oklahoma City, OK, 73104; [2]Department of Ophthalmology/Duke Eye Center, Duke University, Durham, NC 27710; [3]Institute of Human Anatomy and Embryology, Universität Regensburg, 93053 Regensburg, Germany; [4]Division of Ophthalmology, Matsue Red Cross Hospital, Matsue, Shimane 690-8506 Japan

Supplementary Figure 1.

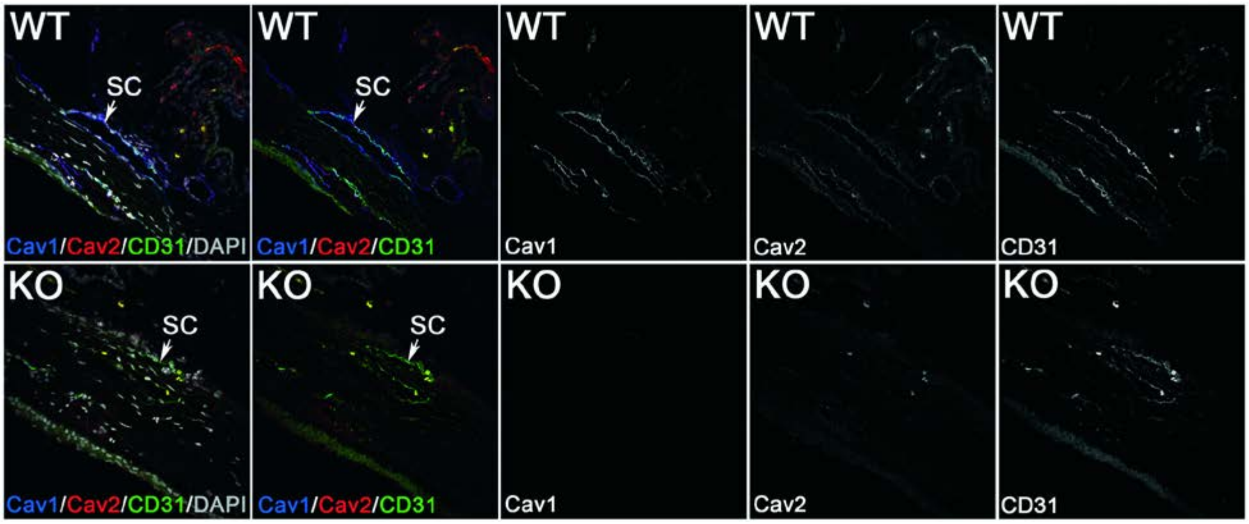

Supplementary Figure 1. Cav-2 immunoreactivity (red in merged images and grayscale in isolated channel) is reduced in *Cav1*$^{-/-}$ outflow pathway. Cav-1 (blue in merged images and grayscale in isolated channels), Cav-2, and CD31 (green in merged images and grayscale in isolated channels) colocalize in Schlemm's canal (SC) in wild-type mice (*upper panels*). Cav-1 is absent and Cav-2 is reduced in the SC of *Cav1*$^{-/-}$ mice demonstrating that Cav-2 cannot compensate for loss of Cav-1.

Supplementary Figure 2.

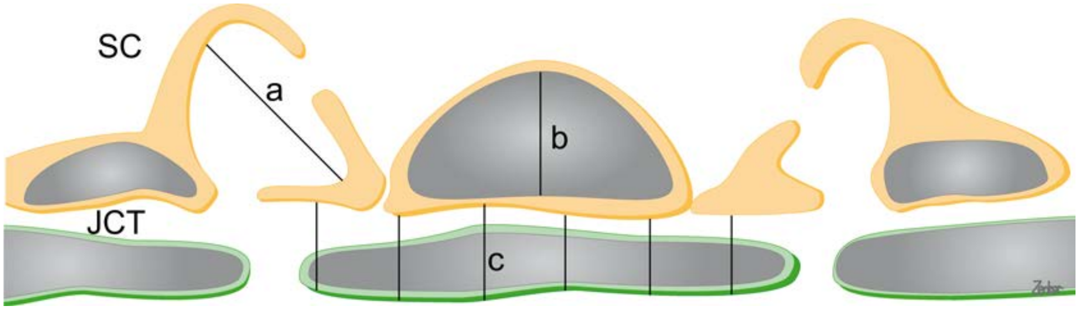

Supplementary Figure 2. Schematic of quantitative transmission electron microscopy analyses. Measurement “a” is GV diameter, “b” is endothelial nuclei thickness, and “c” is juxtacanalicular connective tissue (JCT) depth.
